# Removal of the Eye-ball under Local Anesthesia

**Published:** 1919-01

**Authors:** Alberto G. Garcia

**Affiliations:** 219 East Sixth Street; Austin, Texas


### Removal of the Eyeball Under Local Anesthesia.

---

ALBERTO G GARCIA, M. D., AUSTIN, TEXAS.

Only such points as will make clear the technique about to be described for anesthetizing the eye will be considered in this article. It is assumed that the operation of enuclcation will be performed by one of the methods commonly used for the purpose. Details of anatomy are left undiscussed, though it is well to have a clear knowledge of this before beginning to anesthetize the eye.

The indications and contraindications for this method of anesthetization are the same as those which govern the operation itself. A state of acute inflammation is a positive contraindication. Efforts to reduce this inflammation to a minimum should be made before attempting to operate, regardless of the kind of anesthesia used. Local anesthesia may be used, however, in many cases where the operation is strongly indicated, but cannot be performed without danger under general anesthesia on account of constitutional disturbances. Among such types of cases may be mentioned extreme old age, heart disease, and nephritis.

The technique as developed in this article is suggested by the various methods described by Schleich, Loewenstein, and Braun.

Before sending the patient to the operating room, he should receive a hypodermic injection of morphine grs. 1/4 atropine grs. 1/150. In twenty minutes the patient is quiet and free from all nervous excitement. Two or three drops of cocaine solution 5% are instilled into the eye. In a few minutes the conjunctiva is insensitive. A few drops of the anesthetizing solution, which is a two per cent solution of novocain to each ounce of which has been added twenty drops of adrenalin hydrochloride, 1/1000 solution, are injected subconjunctivally around the cornea. By means of the eye speculum, the external canthus of the eye is drawn outward and slightly downward. Just inside of the canthus in this position a hypodermic needle two and a half inches long is inserted on a plane horizontal to the body and is directed backward and slightly inward to a point a little in front of where the optic nerve enters the optic foramen. Two and a half cubic centigrams of the solution are here injected and the needle removed. The inner canthus is similarly drawn inward and a little upward and the needle inserted just inside of the canthus on a horizontal plane, being directed backward and a little outward toward the optic nerve. Here also two and a half cubic centigrams of the solution are injected and the needle removed. By these two deep injections the optic and ciliary nerves are blocked, permitting the removal of the eyeball without any pain whatever. The operation may be started in about ten to fifteen minutes after the last injection.

Two cases have been anesthetized in this manner and have been operated upon with excellent results. Their clinical histories follow:

Case 1. This was seen in June of 1915. Female, Mexican, age 56. Three days after receiving an injury in the right eye by a flying splinter while she was cutting wood, she presented herself with her right eye collapsed but uninflamed. After applying a wet boric acid dressing over the eye she was directed to come the next morning for an operation. The injured eye was relieved under local anesthesia. Though previous to the operation she seemed somewhat nervous, she passed through the operative ordeal without any pain and in remarkably perfect calm. Three hours after the operation she complained of pain in the eye. This, however, was controlled easily with codeine grs. ¼ given every four hours. The second day she was notably free from pain and was able to be up. On the third day she was permitted to have a full diet and to be up all day. By the fifth day after the operation the wound was completely healed, and on the eighth day she left the hospital.

Case 2. Male, Mexican, age 15. This was first seen July

8, 1918. At the time he was suffering from an acute exacerbation of a chronic iridocyclitis, traumatic in origin. After several weeks' treatment the inflammation had been considerably reduced and an operation was advised. This, however, the parents refused at the time. No more was heard of the case until December 9, when the patient again presented himself prepared to undergo the operation. He was beginning to notice slight pains in the good eye, and the blind eye was shrinking considerably. There was no inflammation in the bad eye. That afternoon I operated under local anesthesia. No pain whatever was experienced during the operation. Repeatedly I asked him during different stages of the operation if he suffered any pain and he always answered in the negative. That evening, about three hours after the operation, he complained of pain in the field of the operation. Codeine sulphate grs. ¼, administered every four hours, however, was sufficient to control this and insure him a fairly good night. By the third day there was no pain whatever and on the seventh he left the hospital entirely well.

Comparing the results obtained under this kind of anesthesia with those obtained under general anesthesia, it may be stated that this method insures complete analgesia in the shortest space of time, with the minimum of drug used, and under the smallest amount of shock. Fifteen minutes usually are sufficient to obtain complete analgesia of the eye and its surrounding parts. With ether or chloroform, on the other hand, one may not be positive just how much of the drug will be necessary to secure complete loss of sensation. The eye is the last to lose its sensation under inhalation anesthesia, and there are some cases who resist during the operation unless they are constantly kept dangerously near the last stage of anesthesia. For this reason some operators prefer chloroform as the anesthetic of choice in this operation in spite of the fact that it is ordinarily a more dangerous drug. Local anesthesia, on the contrary, is absolutely harmless. Two or three grains of novocain generally are sufficient for the operation. The addition of adrenalin hydrochloride materially enhances its anesthetizing properties and maintains analgesia of the parts for about three hours. Though the patient is perfectly conscious during the operation, he remains very calm and thus materially assists with the operation. The blood vessels in the operative field under local anesthesia are blanched so that the operation is practically a bloodless one. This is quite in contrast with what is a fact under general anesthesia. The blood vessels are markedly engorged, and a good deal of oozing interferes with the smooth progress of the operation. The after effects, nausea, vomiting, and headache, so common under general anesthesia, practically are absent under local anesthesia. This permits the patient to return to his ordinary habits of life in the minimum space of time. In conclusion it may be stated that results thus far obtained strongly suggest that local is the preferable method of anesthesia in such extensive operations as the removal of the eyeball.

219 East Sixth Street.

